# Meta-QTL and ortho-MQTL analyses identified genomic regions controlling rice yield, yield-related traits and root architecture under water deficit conditions

**DOI:** 10.1038/s41598-021-86259-2

**Published:** 2021-03-25

**Authors:** Bahman Khahani, Elahe Tavakol, Vahid Shariati, Laura Rossini

**Affiliations:** 1 Department of Plant Genetics and Production, College of Agriculture, Shiraz University, Shiraz, Iran; 2 NIGEB Genome Center, National Institute of Genetic Engineering and Biotechnology, Tehran, Iran; 3 Università degli Studi di Milano-DiSAA, Milan, Italy

**Keywords:** Genetics, Plant sciences

## Abstract

Meta-QTL (MQTL) analysis is a robust approach for genetic dissection of complex quantitative traits. Rice varieties adapted to non-flooded cultivation are highly desirable in breeding programs due to the water deficit global problem. In order to identify stable QTLs for major agronomic traits under water deficit conditions, we performed a comprehensive MQTL analysis on 563 QTLs from 67 rice populations published from 2001 to 2019. Yield and yield-related traits including grain weight, heading date, plant height, tiller number as well as root architecture-related traits including root dry weight, root length, root number, root thickness, the ratio of deep rooting and plant water content under water deficit condition were investigated. A total of 61 stable MQTLs over different genetic backgrounds and environments were identified. The average confidence interval of MQTLs was considerably refined compared to the initial QTLs, resulted in the identification of some well-known functionally characterized genes and several putative novel CGs for investigated traits. Ortho-MQTL mining based on genomic collinearity between rice and maize allowed identification of five ortho-MQTLs between these two cereals. The results can help breeders to improve yield under water deficit conditions.

## Introduction

Rice is the world’s most important staple food and it is an excellent model crop for plant genetic studies^1^. Considering climate change scenarios and increasing water deficits, rice breeding programs have invested significant efforts into producing new rice varieties suitable for growing under reduced water inputs^2–4^. Tolerance to water deficit is a highly complex trait controlled by quantitative trait loci (QTLs). QTL mapping based on bi-parental populations is strongly influenced by the choice of marker sets, parents, population size, population types and environments^5–9^ hampering the transfer of QTLs and associated markers across different breeding programs. A powerful approach to circumvent this issue is Meta-analysis of QTLs (MQTL), which compiles QTL data from independent studies, locations, years and genetic backgrounds in order to detect stable and reliable QTLs^10–12^. An additional benefit of this approach is the reduction of confidence intervals (CIs) in the MQTLs leading to improved genetic resolution for marker-assisted selection (MAS) and identification of candidate genes (CGs). Together, MQTL analysis may increase selection accuracy and efficiency, thus enhancing genetic gains in plants breeding programs^5,9,13–15^. Several MQTL studies for drought stress have been conducted in cereals such as wheat^16^, maize^8,17^, and barley^5,18^. While a recent rice MQTL study considered various traits under unstressed conditions^19^, relatively few reports address water deficit conditions in rice: MQTL studies by Swamy et al. and Trijatmiko et al. focused on yield integrating data from 15 and 13 experiments^20,21^, respectively, and Khowaja et al. and Yang et al. reported some MQTLs for plant height and heading date based on QTLs published until 2009 and 2011, respectively^22,23^.

In the current study, we conducted a comprehensive genome-wide meta-analysis on QTLs reported in the last two decades controlling yield and yield-related traits in rice under water deficit conditions including Yield (YLD), grain weight (GW), heading date (HD), plant height (PH), tiller number (TN) as well as some drought tolerance criteria. Moreover, considering the key role of root architecture in plant responses to water deficit, different root related traits including root dry weight (RDW), root length (RL), root thickness (RT), roots number (RN) and rate of deep rooting (RDR) were subjected to MQTL analyses. We further scanned refined intervals of resulting stable QTLs for CGs related to the aforementioned traits. Additionally, to evaluate transferability of information to other cereals, ortho-MQTLs were investigated based on genomic collinearity between rice and maize^24^. Results will be applicable to improve selection for yield potential, stability and performance under water deficit conditions in cereal breeding programs.

## Results and discussion

### Distribution of yield and yield-related QTLs under water deficit conditions on the rice genome

**Figure 1:**
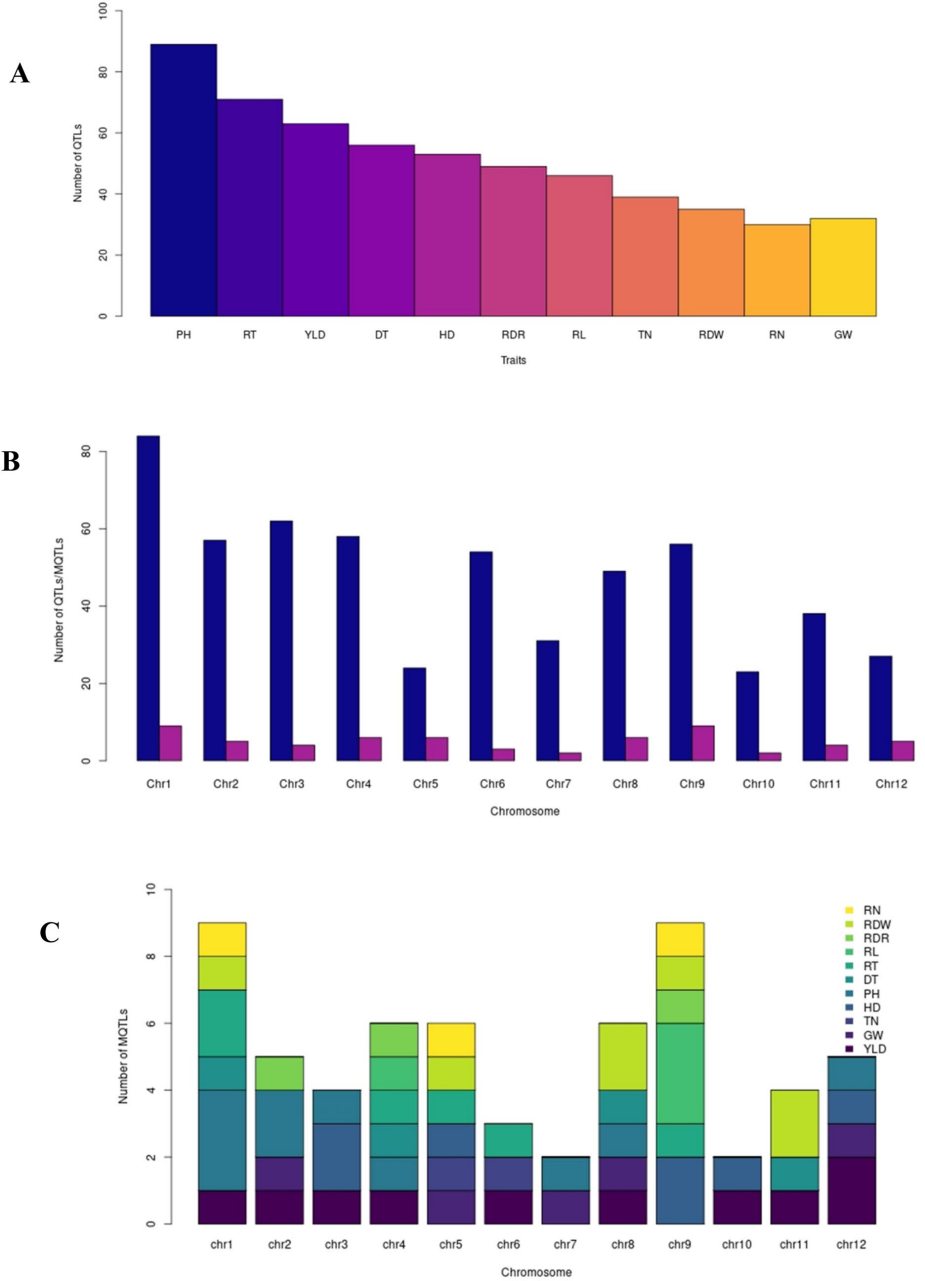
The number and distribution of QTLs and MQTLs. (**A**) The number of initial QTLs used in the MQTL analysis for YLD, HD, PH, GW, TN, DT, RT, RL, RDR, RDW and RN. (**B**) The distribution of QTLs and MQTLs on the twelve chromosomes of rice shown in dark blue and purple, respectively. (**C**) The number of MQTLs for different traits on each chromosome of rice.

In order to discover consensus genomic regions associated with YLD, PH, TN, HD, GW, RDW, RL, RT, RN and RDR and some drought tolerance-related traits including drought response index (DRI), relative water content (RWC), canopy temperature (CT), leaf rolling (LR), leaf drying (LD) under water deficit conditions in rice, we compiled a total of 563 QTLs derived from 67 QTL populations (57 studies) reported from 2001 to 2019 (Table 1; Fig. 1A). The number of QTLs for each trait and their distribution on 12 rice chromosomes are shown in Fig. 1A,B. The chromosome 1 harbored the highest number of QTLs for all studied traits with 84 initial QTLs followed by chromosome 3 (62 QTLs) and chromosome 4 (58 QTLs). Whereas chromosome 10 harbored the lowest number of QTLs with 23 QTLs (Fig. 1B). The distribution of QTLs on different rice chromosomes with the highest number of QTLs on chromosomes 1 and 3 was similar to previous reports^14,19,20^. The number of QTLs on each chromosome exhibited a positive correlation (r = 0.73) with the length of chromosome.

**Table 1:** Summary of QTL studies used in the QTL meta-analysis for YLD, GW, HD, PH, TN, RWC, CT, LR, LD, DRI, RDW, RL, RN, RT, and DT traits in rice under water deficit condition.

Ref no.	Number of QTL population(s)	Parents of population	Population type	Population size	No. of markers	Map density (cM)	Marker type	Trait(s)	References
1	1	Caiapo × IRGC105491	BC	300	718	2.49	SSR, RFLP	HD, PH, GW	^99^
2	1	IR58821 × IR52561	RIL	183	178	5.29	RFLP, AFLP	RT	^100^
3	1	Bala × Aucena	RIL	205	6969	0.20	RFLP, AFLP, SSR	RDW, RT, RL, RN	^101^
4	1	CT9993 × IR62266	DH	220	399	5.49	RFLP, AFLP, SSR	YLD, HD, PH, RWC, CT, LR, LD	^102^
5	1	IAC65 × Co39	RIL	125	115	10.20	RFLP	RL, RT	^103^
6	1	Zhenshan 97 × Minghui 63	RIL	241	208	8.05	SSR, RFLP	YLD, GW	^104^
7	1	Milyang23 × Akihikari	RIL	191	182	6.56	RFLP	TN	^105^
8	1	IR1552 × Azucena	RIL	96	117	11.01	RFLP, AFLP, SSR	RL, RN	^106^
9	1	Zhenshan 97 × Minghui 63	RIL	241	208	8.05	SSR, RFLP	PH, TN, HD	^107^
10	1	CT9993 × IR62266	DH	220	182	4.19	RFLP, AFLP, SSR	YLD, HD, PH	^108^
11	1	Yuefu × IRAT109	DH	116	4662	0.23	SSR, RFLP	RT, RN, RL, RDW	^109^
12	1	ZenShan 97B × IRAT109	RIL	187	339	2.99	SSR	YLD, GW	^2^
13	1	ZenShan 97 × IRAT109	RIL	180	683	2.45	SSR	HD, DRI, LR, LD	^67^
14	1	Bala × Azucena	RIL	177	592	0.58	RFLP, AFLP	PH, HD, LD	^110^
15	1	Akihikari × IRAT109	BC	106	2506	0.23	SSR	RDW, RL	^111^
16	1	IR58821 × IR 52,561	RIL	148	231	5.43	RFLP, AFLP	YLD, GW, PH, HD, CT, LR, LD	^68^
17	1	IR64 × Azucena	BC	323	944	0.27	SSR, RFLP	RL, RT	^112^
18	1	ZenShan 97B × IRAT109	RIL	182	6969	0.20	SSR	RDR, RL	^113^
19	1	Otomemochi × Yumenohatamochi	RIL	98	2187	0.25	SSR	RDW, RN, RL	^114^
20	1	Taichung 189 × Milyang 23	F2	100	718	2.49	SSR	YLD, GW, PH	^115^
21	1	CT9993 × IR62266	DH	220	154	5.14	AFLP	YLD, HD	^116^
22	1	Vandana × Way Rarem	F2	436	112	12.37	SSR	YLD, PH, HD	^117^
23	1	IRAT109 × Yuefu	RIL	120	1541	0.21	SSR	RT	^118^
24	1	Yuefu × IRAT109	RIL	120	6969	0.25	SSR	RT, RN, RL	^119^
25	1	CT9993 × IR62266	DH	220	207	4.96	RFLP, AFLP	YLD, HD, PH, LD, RWC	^69^
26	1	Kinandang Patong × IR64	F2	117	1694	0.20	SSR, STS	RT	^120^
27	1	Zhenshan 97 × IRAT109	RIL	180	344	2.69	SSR	PH	^121^
28	1	IR64 × Azucena	DH	96	110	9.50	RFLP, SSR	RL	^122^
29	1	Norungan × IR64	RIL	380	126	7.61	SSR	YLD, GW, PH, TN, LR, RWC	^31^
30	1	IR20 × Nootripathu	RIL	250	24	14.90	SSR, RAPD, EST	PH, TN, CT, LD, LR	^123^
31	2	Yuefu × IRAT109	BC	430	4475	0.23	SSR	RT	^124^
		Yuefu × IL255	F2	304	7	2.95	SSR	RT	
32	1	CT9993 × IR62266	DH	135	399	5.49	SSR, AFLP, RFLP	YLD, HD, GW, PH, TN, DRI	^3^
33	1	IR64 × INRC10192	RIL	140	14	11.20	SSR	RDW	^125^
34	1	IR64 × Kinandang Patong	RIL	117	406	0.33	SSR, STS	RDR	^126^
35	1	CT9993 × IR20	BC	234	577	0.24	SSR	RT	^127^
36	1	Teqing × Binam	BC	77	718	2.49	SSR	YLD, GW, PH	^128^
37	1	OM1490 × WAB880	BC	229	133	11.06	SSR	YLD, HD, PH	^129^
38	2	HKR47 × MAS26	F2	94	74	11.70	SSR	YLD, PH, TN	^4^
		MASARB25 × Pusa Basmati 1460	F2	100	33	13.26	SSR	YLD, PH, TN	
39	3	Kinandang Patong × ARC5955	F2	138	1307	0.23	SNP, SSR	RDR	^130^
		Kinandang Patong × Pinulupot1	F2	134	577	0.24	SNP, SSR	RDR	
		Kinandang Patong × Tupa729	F2	133	1259	0.22	SNP, SSR	RDR	
40	1	IR64 × Dro1-NIL	BC	4560	406	0.33	SSR	RDR	^131^
41	2	Tarom Molaei × Teqing	BC	85	718	2.49	SSR	YLD, GW	^132^
		Tarom Molaei × IR64	BC	72	718	2.49	SSR	YLD, GW	
42	1	IR77298 × Sabitri	BC	294	68	3.39	SSR	YLD, HD	^38^
43	1	IR55419 × TDK1	BC	365	418	0.68	SSR	YLD, HD, PH	^133^
44	1	Xiaobaijingzi × Kongyu 131	RIL	220	73	12.89	SSR	YLD, PH	^134^
45	3	Kinandang Patong × Momiroman	F2	123	3129	0.20	SNP, SSR	RDR	^135^
		Kinandang Patong × Yumeaoba	F2	128	4749	0.22	SNP, SSR	RDR	
		Kinandang Patong × Tachisugata	F2	121	2923	0.23	SNP, SSR	RDR	
46	1	Yuefu × IRAT109	F2	2013	5	5.48	SSR	RT, RL	^136^
47	1	Zhenshan 97B × IRAT109	RIL	180	3129	0.22	SNP	RDR	^137^
48	3	IR20 × Nootripathu	RIL	397	51	16.79	SSR	PH, RWC, CT, LR	^32^
		IR20 × Nootripathu	RIL	340	51	16.79	SSR	YLD, HD, GW, PH, TN	
		IR20 × Nootripathu	RIL	330	51	16.79	SSR	YLD, PH	
49	1	Nipponbare × Kasalath	F2	155	934	0.38	SSR, RFLP, AFLP	RN	^138^
50	1	Kinandang Patong × IR64	F2	121	1220	0.21	SSR, SNP	RDR	^139^
51	1	KaliAus × AUS276	BC	276	6969	0.20	SNP	RDW, RL	^140^
52	1	IR64 × Dular	RIL	490	1892	0.24	SSR	RL, RDW, RN, RDR	^141^
53	1	N-22 × Cocodrie	RIL	183	2670	0.25	SNP	RL, RDW	^142^
54	1	Cocodrie × Vandana	F2	187	136	7.75	SNP	YLD	^56^
55	2	D123 × Shennong265	BC	178	40	12.24	SSR	GW, PH, HD	^39^
		D123 × Shennong265	BC	314	29	19.04	SSR	YLD, GW, PH, TN	
56	1	IR55419 × Super Basmati	F2	418	1702	0.25	SSR	RDW, RL	^143^
57	1	M-203 × M-206	RIL	241	2474	0.23	SNP	RL, RDW	^144^

*BC* backcross, *DH* double haploids, *RIL* recombinant inbred lines, *YLD* yield, *GW *grain weight, *PH* plant height, *HD* heading date, *TN* tiller number, *RWC* relative water content, *CT* canopy temperature, *LR* leaf rolling, *LD* leaf drying, *DRI* drought response index, *RDW* root dry weight, *RL* root length, *RN* root number, *RT* root thickness, *RDR* ratio of deep rooting.

Among the studied traits, PH, RT and YLD had the highest number of QTLs with 89, 71 and 63 QTLs, respectively (Fig. 1A). The highest number of QTLs for PH was located on chromosome 1 with 24 QTLs, whereas chromosome 3 with 16 and 12 QTLs had the highest number of QTLs for HD and YLD, respectively. The QTLs for TN were mainly situated on chromosome 6 and QTLs for GW were evenly distributed on all chromosomes. For RWC, DRI, CT, LD and LR traits related to drought tolerance, 56 QTLs were distributed all over 12 chromosomes in rice with the highest number of QTLs on chromosome 2.

### Detected MQTLs and their distribution on the rice genome

A total of 527 QTLs out of the 563 initial QTLs (93%) were successfully projected on the reference map (Table 2). Consequently, chromosome 1 had the highest (83) and chromosome 5 and 10 had the lowest (21) number of projected QTLs. The meta-analysis greatly summarized the total number of projected QTLs from 527 to 61 MQTLs (11.5%; Fig. 1B,C) supported by at least two QTLs deduced from different populations and considerably reduced the respective confidence intervals (CI) in comparison to the initial QTLs (Table 3). Therefore, MQTL analysis can efficiently confine the number of QTLs and narrow down the genomic regions controlling different traits^19^.

**Table 2:** The number of initial QTLs on the 12 chromosomes of rice for YLD, GW, HD, PH, TN, DT, RT, RL, RDR, RDW and RN traits under water deficit condition used for MQTL analysis after integrating into the reference map.

Chromosome	PH	RT	RL	YLD	HD	RDR	DT	RDW	TN	RN	GW	Total
1	24	11	4	7	2	3	7	7	7	7	3	83
2	10	4	2	5	2	9	8	1	3	2	3	49
3	5	3	4	12	14	4	5	2	2	5	4	60
4	9	12	8	6	1	11	2	1	1	3	2	56
5	2	2	3	0	2	0	1	3	2	2	4	21
6	9	7	1	10	5	3	3	2	7	3	1	51
7	4	2	1	1	2	5	3	3	2	2	2	27
8	7	6	3	5	5	2	6	4	4	1	3	46
9	3	12	13	0	6	4	6	4	2	2	0	52
10	2	3	1	4	3	5	1	0	2	0	0	21
11	3	4	3	3	3	3	4	6	2	2	4	37
12	5	3	3	5	4	0	1	1	0	1	2	25
Total	83	69	46	58	49	49	47	34	34	30	28	527

*YLD* yield, *GW* grain weight, *PH* plant height, *HD* heading date, *TN* tiller number, *DT* drought tolerance, *RDW* root dry weight, *RL* root length, *RN* root number, *RT* root thickness, *RDR* ratio of deep rooting.

**Table 3:** Summary of the detected MQTLs for YLD, GW, HD, PH, TN, DT, RT, RL, RDR, RDW and RN traits in rice under water deficit condition.

Trait	Chr	MQTL	Flanking markers	Position on the consensus reference map (cM)	Confidence interval (cM)	Genomic position on the rice genome (Mb)	Number of initial QTLs	Number of studies/populations	Phenotypic variance range (%)	Number of genes underlying the MQTL interval
GW	2	MQTL-GW1	RG102-R418	118.97	1.84	27.48–28.94	2	2/2	5.9–10	170
	5	MQTL-GW2	C61983S-RM3419	34.86	2.54	4.33–5.28	4	4/4	8.63–14.2	76
	7	MQTL-GW3	C1467-R10022S	83.43	19.85	20.73–25.43	2	1/2	6.95–8.82	597
	8	MQTL-GW4	S3680-RM3689	79.81	0.96	18.25–19.33	2	2/2	4.15–10	78
	12	MTQL-GW5	R10851S-RM7376	79.84	19.14	19.87–23.44	2	2/2	8–21.9	257
HD	3	MQTL-HD1	C60980S-RM6496	43.96	3.92	8.80–10.14	5	2/2	10.2–22.3	194
	3	MQTL-HD2	S1764-RM6881	80.74	2.93	15.94–16.87	3	2/2	9.3–10.6	70
	5	MQTL-HD3	RM305-RM2357	92.66	33.1	20.94–26.85	2	2/2	8.5–17.15	750
	9	MQTL-HD4	S781-G1047	44.29	0.3	1.21–4.70	2	2/2	9.97–15.8	181
	9	MQTL-HD5	R1751-S2074	94.7	1.71	14.36–15.07	2	2/2	7.03–23.8	73
	10	MQTL-HD6	RM4455-C1369	32.9	22.85	11.66–17.15	3	2/2	3.54–8.06	456
	12	MQTL-HD7	C53024S-RM1337	51.07	0.43	10.60–11.93	4	3/3	5–21.84	66
PH	1	MQTL-PH1	RM8066-RM3627	54.37	3.01	9.56–10.30	3	2/2	5.22–11.48	75
	1	MQTL-PH2	R530-RM3324	129.7	3.07	30.50–31.71	2	2/2	10–22.7	182
	1	MQTL-PH3	RM6387-RM3285	137.42	0.12	32.54–33.04	3	2/3	9.9–27.5	62
	2	MQTL-PH4	S14115-G1340	45.05	6.29	8.72–10.42	2	2/2	5.83–12.3	130
	2	MQTL-PH5	RM208-RM498	140.44	0.03	35.13–35.39	5	2/2	2.9–13.9	46
	3	MQTL-PH6	C52104S-E1419S	92.38	1.27	23.13–23.88	2	2/2	4.62–6.06	58
	4	MQTL-PH7	RG329-RM3836	106.79	1.28	30.85–31.62	6	4/4	2.26–14.4	115
	7	MQTL-PH8	RM3718-R1788	49.37	3.68	7.95–15.20	3	2/2	4.33–4.44	338
	8	MQTL-PH9	RM7049-E60162S	92.7	4.15	20.81–21.76	2	2/2	10–28.2	92
	12	MQTL-PH10	S10904-C53024S	49.59	1.41	7.98–10.60	4	3/3	4.94–13.11	120
TN	5	MQTL-TN1	C1268-S10569	80	5	20.15–20.80	2	2/2	4.19–14.7	76
	6	MQTL-TN2	C1032-RM8258	14.54	2.76	3.16–4.73	2	2/2	9.39–10	230
YLD	1	MQTL-YLD1	RM1152-G1372	127	0.6	30.09–30.49	3	2/2	5–14.57	70
	2	MQTL-YLD2	RM5706-L107	111.56	3.6	26.47–27.59	2	2/2	10–43.2	142
	3	MQTL-YLD3	C51151S-RM3525	131.47	9.37	28.56–30.38	4	2/2	6.35–15	224
	4	MQTL-YLD4	R2737-RG329	100.94	7.3	29.15–30.85	2	2/2	1.31–15.8	229
	6	MQTL-YLD5	RM5531-R10069S	54.48	7.05	7.17–10.46	2	2/2	6.7–12.18	183
	8	MQTL-YLD6	RM2344-RZ143	16.62	5.96	0.07–1.52	2	2/2	3.24–8.5	198
	10	MQTL-YLD7	R1261-C63979S	16.65	0.4	8.85–9.92	2	2/2	9.5–11.4	53
	11	MQTL-YLD8	RM6085-S20163S	28.03	12.3	3.04–5.37	2	2/2	8.5–15.5	219
	12	MQTL-YLD9	E30009S-R3276S	46.69	6.68	6.98–10.43	3	2/2	13.89–30	178
	12	MQTL-YLD10	S10043S-S826	58.47	3.9	15.32–17.56	2	2/2	9.27–22.3	66
DT	1	MQTL-DT1	RM7318-C10728S	113.16	0.41	26.14–26.88	4	3/3	9.25–250.8	89
	4	MQTL-DT2	C12216S- E61747S	41.52	2.5	18.39–18.44	2	2/2	7.7–10.19	24
	8	MQTL-DT3	RM7356-S11114	93.44	0.54	21.28–21.47	2	2/2	10–10.1	21
	11	MQTL-DT4	E20817-E3558S	74	2.71	16.81–17.89	2	2/2	19.05–19.8	74
RT	1	MQTL-RT1	C409-RM7566	111.11	11.93	24.93–27.76	2	2/2	8.7–10.1	308
	1	MQTL-RT2	E50125S-RM5759	150.68	6.05	37.23–39.02	2	2/2	7.2–21	291
	4	MQTL-RT3	C1087-C377	70.17	7.01	21.98–23.99	2	2/2	7.7–20.6	294
	5	MQTL-RT4	RM3381-RM5948	62.56	16.45	9.58–18.97	2	2/2	5–7.4	546
	6	MQTL-RT5	RM8112-RM584	13.32	6.98	2.17–3.41	2	2/2	2–10.8	214
	9	MQTL-RT6	RM3787-C482	123.47	3.01	20.04–21.05	4	2/2	10.9–14.6	155
RL	4	MQTL-RL1	RM6992-RM6909	105.97	6.41	30.76–32.06	2	2/2	8.45–11.86	194
	9	MQTL-RL2	C2985-C397	81.42	3.07	11.79–12.28	6	3/3	9.11–11	47
	9	MQTL-RL3	S4677S-RM6839	92.16	3.33	13.62–14.56	4	4/4	8.2–32.5	91
	9	MQTL-RL4	C12357S-RM6643	132.34	0.58	21.52–21.70	2	2/2	12.9–13.4	35
RDR	2	MQTL-RDR1	R418-RM6424	124.07	2.14	28.94–29.62	4	2/3	9.3–19.9	88
	4	MQTL-RDR2	RM5320-R2737	91.65	8.52	28.01–29.15	4	3/3	10–56.6	133
	9	MQTL-RDR3	RM5526-RM7038	78.11	4.22	7.31–11.80	2	2/2	7.99–10	220
RDW	1	MQTL-RDW1	E50125S-RM6593	148.37	0.95	37.23–38.02	3	3/3	7.6–26.8	134
	5	MQTL-RDW2	E417S-RM3631	104.43	3.42	24.11–25.83	2	2/2	10–12.2	214
	8	MQTL-RDW3	RM8266-RM8256	53.06	12.21	3.98–7.78	2	2/2	3.3–7.9	294
	8	MQTL-RDW4	S11102-RM8043	103.72	1.56	22.87–23.57	2	2/2	4.4–16	72
	9	MQTL-RDW5	RM3909-C11503S	120.57	1.19	19.53–19.88	2	2/2	4.31–13.1	51
	11	MQTL-RDW6	S2137-C61883S	57.03	8.53	8.29–10.13	2	2/2	6.14–14	94
	11	MQTL-RDW7	RM7240-RM6688	119.95	0.25	27.02–27.54	4	3/3	2.2–11.1	46
RN	1	MQTL-RN1	RM2772-C808	104.4	3.38	24.08–25.63	2	2/2	12–22.8	182
	5	MQTL-RN2	RM5401-RM2457	100.8	16.25	22.28–26.87	2	2/2	5.1–10	602
	9	MQTL-RN3	RM3808-C482	124.14	2.2	20.54–21.05	2	2/2	8.6–11.6	75

*YLD* yield, *GW* grain weight, *PH* plant height, *HD* heading date, *TN* Tiller number, *DT* drought tolerance, *RDW* root dry weight, *RL* root length, *RN* root number, *RT* root thickness, *RDR* ratio of deep rooting, *Chr* chromosome.

**Figure 2:**
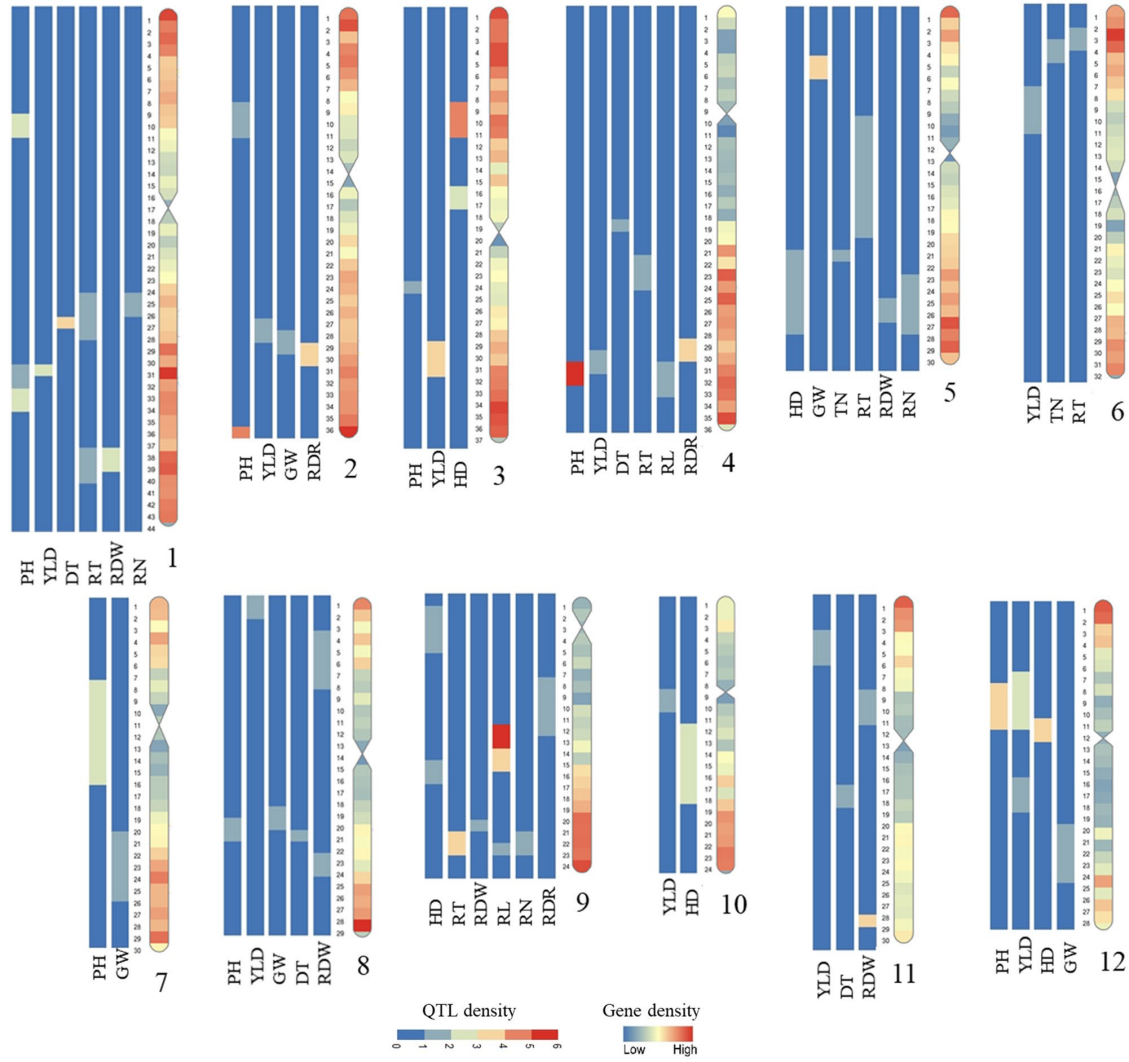
Heatmap of MQTLs for YLD, HD, PH, GW, TN, DT, RT, RL, RDR, RDW and RN presented on the rice genome in Mb. The gene density of each chromosome is indicated on the right chromosome.

The number of MQTLs per chromosome ranged from two (chromosome 10) to nine (chromosomes 1 and 9) with an average of 5.08 MQTLs (Fig. 2; Table 3; Additional file 1). Chromosomes 1 and 9 with nine MQTLs and chromosomes 10 and 7 with two MQTLs had the highest and lowest number of MQTLs, respectively (Table 3; Additional file 1). There was a low correlation between the number of initial QTLs and the final number of MQTLs on each chromosome (r = 0.58).

Out of the total number of 61 MQTLs, we detected 10 MQTLs for YLD, five MQTLs for GW, seven MQTLs for HD, 10 MQTLs for PH, two MQTLs for TN, four MQTLs for DT, seven MQTLs for RDW, six MQTLs for RT, four MQTLs for RL, three MQTLs for RN and three MQTLs for RDR. These MQTLs were stable across different environments and genetic backgrounds. MQTL-PH7 and MQTL-RL2 involving the highest number of initial QTLs (6) were considered as the most stable QTLs (Table 3; Additional file 1). Among the identified MQTLs, four MQTLs for HD (MQTL-HD3, MQTL-HD4, MQTL-HD5 and MQTL-HD6) and three MQTLs for YLD (MQTL-YLD2, MQTL-YLD7 and MQTL-YLD10) overlapped with previously reported MQTLs under drought conditions in rice^20,23^. To the best of our knowledge this is the first MQTL analysis for GW, TN and DT in rice.

**Figure 3:**
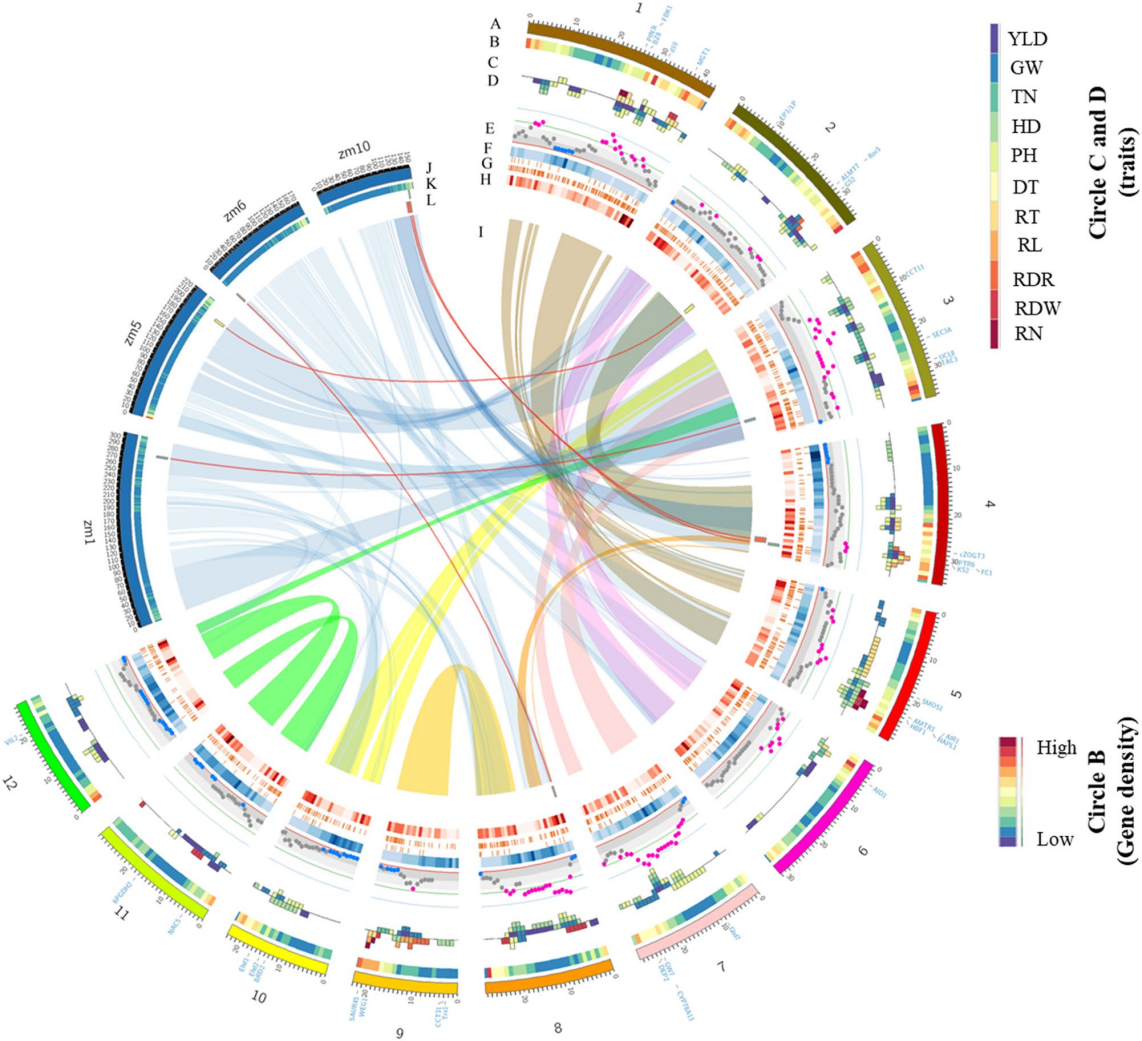
The distribution pattern of (**A**) functionally characterized genes on rice chromosomes, (**B**) gene density on rice chromosomes, (**C**) MQTLs under water deficit condition for different traits indicated in the color scale on the right side, (**D**) MQTLs under normal condition for different traits indicated in the color scale on the right side, (**E**) QTLs density, (**F**) SNP density shown in white to dark blue scale for the lowest to the highest density, (**G**) Structural variants (SV) density shown in white to dark red scale for the lowest to the highest density, (**H**) recombination density shown in white to dark red scale for the lowest to the highest density, (**I**) rice duplicated regions and rice syntenic regions with maize in light blue, (**J**) maize chromosomes with orthologous MQTLs with rice, (**K**) gene density on the maize chromosomes and (**L**) ortho-MQTLs between rice and maize. The outermost circle represents the rice genome in Mb.

A total of 10 MQTLs were detected in the same chromosomal regions with similar yield and yield-related traits under well-water condition in rice^19^. This indicates the same loci might control aforementioned traits under both water deficit and well-water conditions (Additional file 2). They include five MQTLs for PH (MQTL-PH2, PH4, PH7, PH8 and PH9) on chromosomes 1, 2, 4, 7 and 8, two MQTLs for GW (MQTL-GW4 and GW5) on chromosomes 8 and 12, two MQTLs for HD (MQTL-HD1 and HD3) on chromosomes 3 and 5 and one MQTL for YLD (MQTL-YLD3) on chromosome 3 (Fig. 3; Additional file 2).

The MQTL analysis considerably narrowed the CI allowing for exploration of a reduced number of candidate genes (CGs) for the investigated traits. The average CI was reduced from 15.57 cM in the initial QTLs to 5.48 cM in the MQTL with 65% of MQTLs having CI < 5 cM (Table 3). In 10 MQTLs, MQTL-GW4, HD4, HD7, PH3, PH5, YLD1, YLD7, DT1, DT3, RL4, RDW1 and RDW7, the CI was reduced to < 1 cM (Table 3). Therefore, MQTL analysis can significantly raise the accuracy of identification of CGs. All the annotated genes located within the CI of each MQTL and the most promising CGs based on their reported function in previous studies are reported in Additional file 3. Some functionally characterized genes such as *GRAIN SIZE 2* (*GS2*), *GRAIN WEIGHT 7* (*GW7*), *Early heading date 1* (*Ehd1*), *DWARF 10* (*d10*) and *Grain number, plant height, heading date7* (*Ghd7*), *OsPIN3t*, *OsSAUR45*, and *WEG1* were located within MQTL-GW1, GW3, HD6, PH2, PH8, RT1, RL4, RDW5, respectively, and *OsAIR1* located at MQTL-RN2 and RDW2, and *OsMGT1* located at MQTL-RT2 and RDW1, that are assumed to control the aforementioned traits. Putative novels CGs for each trait are discussed below. In addition, the positions of MQTLs on the rice genome were compared with the gene density, and densities of SNPs, structural variants (SV), recombination and functional variants, and the reported selective sweep regions^25^ (Fig. 3). Most of the detected MQTLs were located in sub-telomeric regions where generally the gene, SNP, SV and recombination densities are higher (Figs. 2, 3). This is consistent with previous results in barley, maize and rice^15,19,26^. The regions with high SV frequency could play an effective role in stress response^27^. A total of 13 MQTLs (MQTL-YLD3, YLD6, GW2, GW5, HD5, TN2, DT2, RT3, RT4, RL3, RL4, RDR3 and RDW6) were co-located with selective sweep regions reported by Huang et al. These MQTLs are likely effective for selection towards drought adaptation during rice breeding and domestication processes^25^. Five of these MQTLs including MQTL-YLD3, TN2, GW5, RT4 and RDW6 were also co-located with the position of reported functional variants^25^.

The investigation of collinear regions within the rice genome resulted in identification of five duplicated regions containing MQTLs for the same traits. MQTL-YLD2 and MQTL-YLD4 on chromosomes 2 and 4, and MQTL-YLD8 and MQTL-YLD9 on chromosomes 11 and 12 for yield, MQTL-RWD1 and MQTL-RWD2 on chromosomes 1 and 5, MQTL-RWD4 and MQTL-RWD5 on chromosomes 8 and 9, and MQTL-RN1 and MQTL-RN2 on chromosomes 1 and 5 for root-related traits are co-located at rice genome duplicated regions (Fig. 3). Duplicated genomic regions derived from common ancestors might contain paralogous genes with similar functions that can be considered as promising CGs controlling the trait^28^. Consequently, we carefully surveyed these regions for detecting possible paralogous CGs in the duplicated regions. In MQTL-RN2, we note the *OsABIL3* or *PP2C50* gene which has a key role in root architecture and response to drought stress by affecting ABA signaling: overexpression of this gene was reported to lead to the ABA insensitivity along with stomatal density and root architecture^29^. The paralogous gene *Os01g0618200* encoding *PP2C07* is also present at the duplicated region on chromosome 1 with MQTL-RN1 for the same root number trait. Moreover, at MQTL-YLD4 interval on chromosome 4, we detected *GRAS23* that contributes to drought response in rice^30^, with paralogues *HAM1* and *HAM2* colocalizing with the duplicated genome on chromosome 2 with MQTL-YLD2 for the yield under drought stress.

### MQTLs and CGs for grain weight

GW is one of the most important components of YLD in rice^1,31^ and it critically limits YLD during late season drought stress^3,32,33^. A total of five MQTLs were identified for GW (Table 3). MQTL-GW2 on chromosome 5 is the most stable MQTL for GW with the highest number of initial QTLs from four independent studies. Among the identified MQTLs for GW, MQTL-GW4 and GW5 on chromosomes 8 and 12 were located at the same region of GW MQTLs under well-water conditions^19^. Therefore, the same genes might control GW under both conditions. MQTL-GW5 and GW2 were co-located with selective sweep regions and functional variants were reported on the former. These MQTLs could be effective for selection towards drought adaptation^25^. The same source of favorable allele from ‘Tarom Molaei’ parent derived from two independent populations was detected in QTLs located at MQTL-GW3 (Additional file 4).

Some well-known genes controlling GW such as *GS2*^34^ and *GW7*^35^ were located within MQTL-GW1 and MQTL-GW3, respectively, suggesting that these genes may play the same role under water deficit conditions. The list of functionally characterized and novel CGs within each MQTL interval are reported (Additional file 3). For instance, the genomic region spanning MQTL-GW3 contains the *CYP78A13*^36^ and *DEP2*^31^ genes that are reported to control grain size and YLD in rice. MQTL-GW4 on chromosome 8 and MQTL-GW5 on chromosome 12 contain *Os08g0390000* encoding brassinosteroid receptor kinase (BRI1)^31^ and the *OsVIL2*^37^ genes, respectively, which regulate grain size in rice.

### MQTLs and CGs for heading date

It is well known that HD is highly correlated with YLD^38^ and drought adaptation^39,40^. We detected seven MQTLs for HD under water deficit conditions including two MQTLs on chromosomes 3 and 9 and one MQTL each on chromosomes 5, 10 and 12 (Table 3). MQTL-HD1 on chromosome 3 had the highest number of supporting QTLs with five QTLs from two independent studies (Table 3).

Among annotated genes within MQTL-HD1, MQTL-HD3 and MQTL-HD6 intervals, *OsCCT11*^41^, *HBF1*^42^ and *Ehd1*^38,43,44^, respectively, were identified as potential candidates for HD under water deficit conditions. *OsCCT11* is considered as a positive regulator of heading date since RNAi-mediated downregulation of this gene delays HD^41^, while *HBF1* is considered as a negative regulator of HD since mutation promotes flowering^42^. Among genes within the MQTL-HD3 interval, basic region/leucine zipper motif (bZIP), *FT-like* and circadian clock genes are promising candidates^43,45,46^. Another CG at this MQTL is *OsHAPL1*, known to prevent flowering under long-day conditions^47^. *OsTrx1*^48^ and *OsCCT31*^41^ are potential candidates for MQTL-HD4. In MQTL-HD6, the *BRD2* gene^49^ and the *Ehd2* and *Ehd1* genes^38,43,45^ are reported to modify flowering time in rice.

### MQTLs and CGs for plant height

Since the Green Revolution, PH has been considered as a major target for YLD improvement^50^ and it also contributes to drought tolerance^40^. Among the studied traits, PH and YLD had the highest number of MQTLs; we identified 10 MQTLs for PH including three MQTLs on chromosome 1, two MQTLs on chromosome 2 and one MQTL each on chromosomes 3, 4, 7, 8 and 12. The MQTL-PH7 on chromosome 4 had the largest number of initial QTLs with six QTLs from four independent studies followed by MQTL-PH10 on chromosome 12 with three QTLs reported from three independent studies. These MQTLs are the most stable QTLs for PH under water deficit conditions.

The *d10*^51^ and *Ghd7*^52^ genes are reported to regulate plant height in rice, and they are positioned within MQTL-PH2 and MQTL-PH8 genomic regions, respectively. MQTL-PH4 contains *EP3/LP* gene, whose mutant shows increased panicle size and PH in rice^53^. Conversely, mutations in *OsKS2* and *NAL1*^54,55^ at MQTL-PH7 and *OsSEC3A*^56^ at MQTL-PH6 decrease PH in rice. In MQTL-PH10, we detected *Os12g0271600* that encodes *BRI1* and the mutant alleles could act as a dwarfism gene^50^.

### MQTLs and CGs for yield

The maintenance of YLD under drought condition is the ultimate goal in cereal breeding^57,58^. We identified 10 MQTLs for YLD consisting of two MQTLs on chromosome 12 and one MQTL each on chromosomes 1, 2, 3, 4, 6, 8, 10 and 11 (Table 3). Among them MQTL-YLD3 on chromosome 3 overlapped with a YLD MQTL identified under well-water conditions^19^. Therefore, the same genes might control YLD under both mentioned conditions at this position.

We detected some genes which affect the photosynthetic rate including *Roc5* at MQTL-YLD2^59^, *UCL8* at MQTL-YLD3^60^ and *OsPTR6* at MQTL-YLD4^61^ that might indirectly contribute to the final YLD. *OsALMT7* is located at the MQTL-YLD2 interval with pleiotropic effects on YLD and panicle size^62^. *TAC3* might indirectly regulate YLD through changing HD and tiller angle in rice at MQTL-YLD3^63^. The most likely CGs at MQTL-YLD5 are *Os06g0274500* which encodes SERK-like gene and BRI1-associated receptor kinase 1 (*BAK1*) that affects grain size and number in rice^64^. The *OsNAC5* gene on MQTL-YLD8 is known to have a positive effect on YLD under drought condition^65^.

### MQTLs and CGs for number of tillers

The number of fertile tillers is a major contributor to YLD and its alteration during drought stress can result in drought adaptation^4,66–68^. Tillering is a complex process and highly affected by environmental conditions^66^. We detected only two MQTLs on chromosomes 5 and 6 which were associated with TN (Table 3). In MQTL-TN2, we identified *OsAID1* as a gene associated to TN regulation in rice^69^.

### MQTLs and CGs for drought tolerance

Plant water content is highly affected by water deficit conditions and in turn can contribute to drought tolerance. Plant water content can be measured by different criteria including RWC, CT, LR, LD and DRI^3,32,33,70,71^. We identified four MQTLs for DT on chromosomes 1, 4, 8 and 11. MQTL-DT1 on chromosome 1 is the most stable MQTL related to rice water content under water deficit conditions with the highest number of initial QTLs (4) from three independent studies. Within the MQTL-DT1 and MQTL-DT4 intervals, we detected several CGs including *OsBZ8*^72^ and *Os6PGDH2*^73^ that contribute to abiotic stresses tolerance, respectively.

### MQTLs and CGs for root architecture

Root architecture develops through dynamic processes that effectively contribute to water deficit adaptation allowing water and nutrient uptake from deep soil^74,75^. We studied five major traits related to root architecture including RDW, RL, RN, RT and RDR under water deficit conditions and identified 23 MQTLs including seven MQTLs for RDW, four MQTLs for RL, three MQTLs for RN, six MQTLs for RT and three MQTLs for RDR (Table 3). MQTL-RL2 had the highest number of initial QTLs (six QTLs from three independent studies) and it was considered as the most stable QTL for root architecture (Table 3). Interestingly, MQTL-YLD4 for YLD under water deficit conditions on chromosome 4 overlapped with MQTL-RDR2 and RL1 (Fig. 2).

Overlapping MQTLs for different root architecture traits included MQTL-RDW1 and MQTL-RT2 on chromosome 1, MQTL-RDW2 and MQTL-RN2 on chromosome 5 and MQTL-RN1 and MQTL-RT1 on chromosome 1, suggesting the possible existence of genes with pleiotropic effects on these traits. For example, the genomic region spanning MQTL-RN2 and MQTL-RDW2 harbors *OsAIR1,* a gene affecting root architecture and contributing to drought tolerance^76^. In the overlap region between MQTL-RT2 and MQTL-RDW1, noteworthy is *OsMGT1* which was shown to affect root architecture during salinity stress^77^. MQTL-RT1 contains *OsPIN3t*^78^ and *OsFBK1*^79^ genes that are reported to control root architecture under water deficit conditions. The *SMOS1* gene within MQTL-RT4 determines root meristem size^80^, and the *cZOGT3*^81^ gene within MQTL-RDR2 regulates root architecture. Additionally, *AMTR1* (MQTL-RN2) affects root architecture under drought stress^82^.

The same source of favorable allele from ‘IRAT109’ parent derived from two independent populations was identified in QTLs located at MQTL-RL3 (Additional file 4). The *FC1* gene^83^ on MQTL-RL1 controls root growth and might contribute to drought tolerance under water deficit conditions. Within MQTL-RL4, we detected a cluster of *small auxin-up RNA* (*SAUR*) genes. Over-expression of *OsSAUR45* regulates root length and other related root traits^84^.

For RDW, we detected two co-located MQTLs including MQTL-RDW1 and RDW2 co-locating with *OsMGT1* and *OsAIR1*genes, respectively. Additionally, *WEG1* at MQTL-RDW5 is a novel gene that regulates root related traits^85^ and may keep the same role under water deficit conditions.

### Ortho-MQTL mining

**Figure 4:**
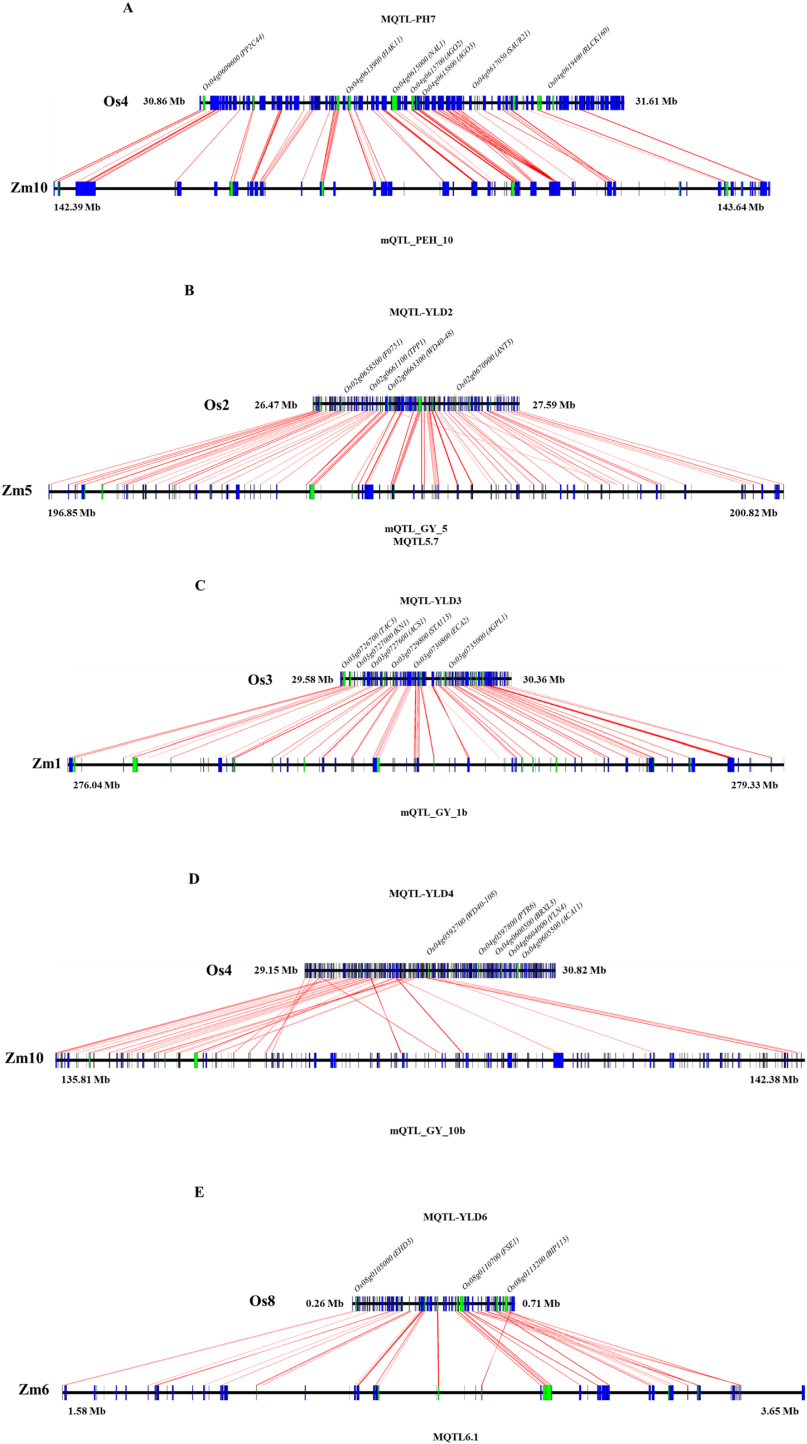
Comparative maps of ortho-MQTLs between rice and maize. (**A**) ortho-MQTL-PH7, (**B**) ortho-MQTL-YLD2, (**C**) ortho-MQTL-YLD3, (**D**) ortho-MQTL-YLD4 and (**E**) ortho-MQTL-YLD6. The chromosome number, genomic position in Mb and the original name of MQTLs are indicated. The orthologous genes in rice and maize are indicated in green color with the corresponding rice gene name.

To investigate ortho-MQTLs for yield and yield-related traits under water deficit conditions between rice and maize as the two most important cereals with generally high water demand, the syntenic regions of all detected rice MQTLs in this study were compared with published maize MQTLs^17,86,87^. Comparative genomic analyses provide a valuable approach to transfer information across species and identify conserved genes^19^. Through synteny analysis between rice and maize, we uncovered 5 ortho-MQTLs including 4 ortho-MQTLs for YLD on chromosomes 2, 3, 4 and 8 and 1 ortho-MQTL for PH on chromosome 4 (Table 4; Fig. 3). The genes located at these syntenic regions were further investigated (Additional file 5; Fig. 4).

**Table 4:** Ortho-MQTLs in rice and maize based on the syntenic analyses.

Ortho-MQTL	Rice MQTL	Rice chr. no. (genomic position in Mb)	Maize original MQTL name	Maize chr. no. (genomic position in Mb)	Maize MQTL reference
Ortho-MQTL-PH7	MQTL-PH7	4 (30.86–31.61)	mQTL_PEH_10	10 (142.39–143.64)	^85^
	MQTL-YLD2	2 (26.47–27.59)	mQTL_GY_5	5 (196.85–199.69)	^84^
Ortho-MQTL-YLD2			MQTL5.7	5 (199.95–200.82)	^16^
Ortho-MQTL-YLD3	MQTL-YLD3	3 (29.58–30.36)	mQTL_GY_1b	1 (276.04–279.33)	^84^
Ortho-MQTL-YLD4	MQTL-YLD4	4 (29.15–30.82)	mQTL_GY_10b	10 (135.81–142.38)	^84^
Ortho-MQTL-YLD6	MQTL-YLD6	8 (0.26–0.71)	MQTL6.1	6 (1.58–3.65)	^16^

MQTL-PH7 and MQTL-YLD4 on chromosome 4 of rice were co-linear with mQTL_PEH_10 and mQTL_GY_10b on chromosome 10 in maize, respectively (Table 4). Three rice MQTLs including MQTL-YLD2, YLD3 and YLD6 on chromosomes 2, 3 and 8, respectively, were situated in syntenic regions of maize yield MQTLs on chromosome 5, 1 and 6, respectively (Table 4; Fig. 3).

The orthologous genes located at these ortho-MQTLs in both rice and maize are shown in the Additional file 5 and Fig. 4. The rice genomic region subtending MQTL-PH7 harbors the *NAL1* gene as a regulator of PH^55^: we identified the ortholog of this gene (*Zm00001d026296*) in the maize ortho-MQTL. In the syntenic region of rice MQTL-YLD2 on chromosome 5 of maize, there were two MQTLs (mQTL_GY_5^86^, MQTL5.7^17^) containing the orthologs of *OsALMT7* and *SID1* genes (*Zm00001d017571* and *Zm00001d017560*), known to affect YLD in rice^62,88^. The orthologous gene of *TAC3* in maize (*Zm00001d033857*) in the syntenic region of MQTL-YLD3 in maize (mQTL_GY_1b) regulates tiller angle that might affect YLD under water deficit conditions^63^. In the syntenic region of rice MQTL-YLD6, there was a MQTL (MQTL6.1^17^) on chromosome 6 of maize. This rice MQTL contains *Ehd3* gene regulating flowering and consequently YLD^38^ and its orthologous (*Zm00001d035008*) was detected in its ortho-MQTL in maize, likely to have similar functions. This approach provided better understanding of genes controlling investigated traits under water deficit conditions with similar evolutionary history and conserved function between these cereals. These results can benefits breeders by tracing CGs and using marker-assisted selection in breeding programs of cereals under water deficit conditions.

## Conclusions

Through MQTL analysis this study provides an overview of genomic regions controlling YLD, yield-related traits, root architecture and plant water content including GW, HD, PH, TN, RDW, RL, RT, RN, RDR and DT under water deficit conditions in rice. This approach is useful in overcoming some limitations of single QTL mapping studies on different genetic backgrounds and environments and greatly facilitates the identification of CGs and robust flanking markers for MAS in breeding programs. The results offer a framework for future genetic studies of yield under drought conditions, e.g. through fine mapping, positional cloning, producing chromosome substitution lines, as well as validation of CGs by genome editing using Clustered Regularly Interspaced Short Palindromic Repeats (CRISPR) and similar approaches. This study also demonstrates the value of ortho-MQTL mining among evolutionarily close crop species for identification of genomic regions and CGs controlling complex quantitative traits.

## Materials and methods

### QTL studies used for MQTL analysis

An exhaustive bibliographic review was carried out on rice QTLs related to yield and yield-related traits under water deficit conditions published from 2001 to 2019. All QTL studies except those lacking proper genetic map information or QTL-related information were used in the MQTL analysis. Consequently, a total of 563 QTLs for YLD, PH, TN, HD, GW, RDW, RL, RT, RN, RDR and traits related to water content of plant under water deficit conditions including DRI, RWC, CT, LR and LD from 67 biparental rice populationsextracted from 57 studies, including all the five major subpopulations of rice—Indica, tropical japonica, temperate japonica, aus, aromatic and also one wild species *O. rufipogon* IRGC 105491 and landraces, were implemented for the MQTL analysis (Table 1). The size of mapping populations varied from 72 to 4560 progenies of various types including 7 DH, 17 F_2_, 15 BC and 28 RIL populations phenotyped in different locations and years (Table 1). Moreover, 56 QTLs related to water content of plant under water deficit conditions including DRI, RWC, CT, LR, LD were subjected to MQTL analysis and resulting MQTLs were reported as drought tolerance (DT). Detailed information on the used QTLs including parents, population type and size, number of markers, map density and evaluated traits are reported in Table 1.

### Projection of QTLs on the reference map

A rice reference map of Wu et al. which is the most comprehensive available genetic map integrated from six identified and saturated maps in rice was chosen based on its high marker density and inclusion of different marker types including SSR, RFLP and AFLP markers. It consists of 6969 markers with an average distance of 0.25 cM between markers, and the average chromosome length is 147.65 cM for a total length of 1771.8 cM^89^. In order to incorporate SNP markers of those initial QTLs with SNP markers (Table 1) into the reference map, we applied our previous approach^19^ in which the genomic position of SNP markers on the rice genome were determined and in consequence the closest markers based on the physical position were used to project them on the reference map.

QTL position, CI, proportion of phenotypic variance (R^2^), log of odds ratio (LOD score), additive effects and favorable alleles were compiled for each QTL in the 67 populations (Additional file 4). In order to calculate 95% CI for QTLs, we used the following formulas: CI = 530/(N × R^2^) for BC and F_2_ lines, CI = 287/(N × R^2^) for DH lines and CI = 163/(N × R^2^) for RLLs lines^90,91^, where N is the population size and R^2^ is the proportion of phenotypic variance of the QTL. MQTL analysis was carried out using BioMercator V4.2^11,92^.

### Meta-QTL analysis and ortho-MQTL mining

The MQTL analysis was conducted on integrated and re-positioned QTLs on the reference map using BioMercator V4.2^11,12,92^. The best model of MQTLs was chosen according to the prevalent value among Akaike Information Criterion (AIC), corrected Akaike Information Criterion (AICc and AIC3), Bayesian Information Criterion (BIC) and Average Weight of Evidence (AWE) criteria. Therefore, the consensus QTL from the best model was reported as a “real” QTL/MQTL^12,92^. Considering the known correlations among RWC, CT, LR, LD and DRI^3,32,33,70^, the respective QTLs were analyzed together as one trait named as DT in BioMercator V4.2.^12,92^. Mapchart V.2.32 software^93^ was used to show the MQTLs and related QTLs on the reference map.

The distribution of MQTLs on the rice genome (IRGSP-1.0) compared to the position of centromeric and telomeric regions and the gene density along each chromosome were surveyed and shown by heatmap using *pheatmap* and R^94,95^. Centromere position, gene density, SNP and structural variations (SV) and recombination rate density of each chromosome, as well as rice genome duplications were retrieved from EnsemblPlants (https://plants.ensembl.org/index.html) database. Additionally, the position of identified MQTLs were compared with selective sweep regions and functional variants in coding regions with strong alteration in allele frequency between cultivated and wild rice reported by Huang et al.^25^. The distribution of aforementioned factors, number of MQTL under water deficit conditions and number of MQTLs under well-water conditions^19^ over the rice genome were shown by using Circos^96^.

To detect ortho-MQTLs between rice and maize, syntenic regions between the two species were identified by using EnsemblPlants database^97^. MQTLs identified for yield and yield-related traits under drought conditions in maize^17,86,87^ were compared with MQTLs detected for similar traits in our study.

### Identification of candidate genes

CGs related to YLD, PH, TN, HD, GW, RDW, RL, RT, RN, RDR and DT traits located in the CI of each detected MQTL were investigated on the rice genome (IRGSP-1.0) using EnsemblPlants and Gramene (http://archive.gramene.org/qtl/). In case of flanking markers without genomic positions, the closest markers were applied for detecting the genomic coordinates of MQTL. Gene annotations within MQTL genomic regions were carefully explored by EnsemblPlants (https://plants.ensembl.org/index.html) and FunRiceGenes (https://funricegenes.github.io/)^98^ databases.

## Supplementary Information

Supplementary Information 1.

Supplementary Information 2.

Supplementary Information 3.

Supplementary Information 4.

Supplementary Information 5.

## Data Availability

The relevant data and additional information are available in the supplementary files and also from the corresponding author on reasonable request.

## Abbreviations

AIC: Akaike information content
AICs: AIC correction
AIC3: AIC 3 candidate models
AWE: Average weight of evidence
BIC: Bayesian information criterion
CGs: Candidate genes
CT: Canopy temperature
CI: Confidence interval
DRI: Drought response index
DT: Drought tolerance
GW: Grain weight
HD: Heading date
LOD: The log of odds ratio
LR: Leaf rolling
LD: Leaf drying
MQTL: Meta-QTL
MAS: Marker-assisted selection
PH: Plant height
QTL: Quantitative trait loci
R^2^: The proportion of phenotype variance
RWC: Relative water content
RDW: Root dry weight
RL: Root length
RT: Root thickness
RN: Roots number
RDR: Rate of deep rooting
TN: Tiller number
YLD: Yield
