# Supplementary material for: Meta-QTL and ortho-MQTL analyses identified genomic regions controlling rice yield, yield-related traits and root architecture under water deficit conditions
Source: Sci Rep. 2021 Mar 25;11:6942. doi: 10.1038/s41598-021-86259-2 (PMC7994909; doi:10.1038/s41598-021-86259-2)
Supplement: Supplementary file 1 — Supplementary Information 1. [file 41598_2021_86259_MOESM1_ESM.docx]

**Comprehensive Meta-QTL and ortho-MQTL analyses identified genomic regions controlling rice yield, yield-related traits and root architecture under water deficit conditions**

Bahman Khahani^1^, Elahe Tavakol^1*^, Vahid Shariati ^2*^, Laura Rossini^3^

^1^ Department of Plant Genetics and Production, College of Agriculture, Shiraz University, Shiraz, Iran.

^2^ NIGEB Genome Center, National Institute of genetic Engineering and Biotechnology, Tehran, Iran.

^3^ Università degli Studi di Milano – DiSAA, Milano, Italy

^*^ Corresponding Authors

E-mail: [elahetavackol@gmail.com](mailto:elahetavackol@gmail.com), vshariati@nigeb.ac.ir


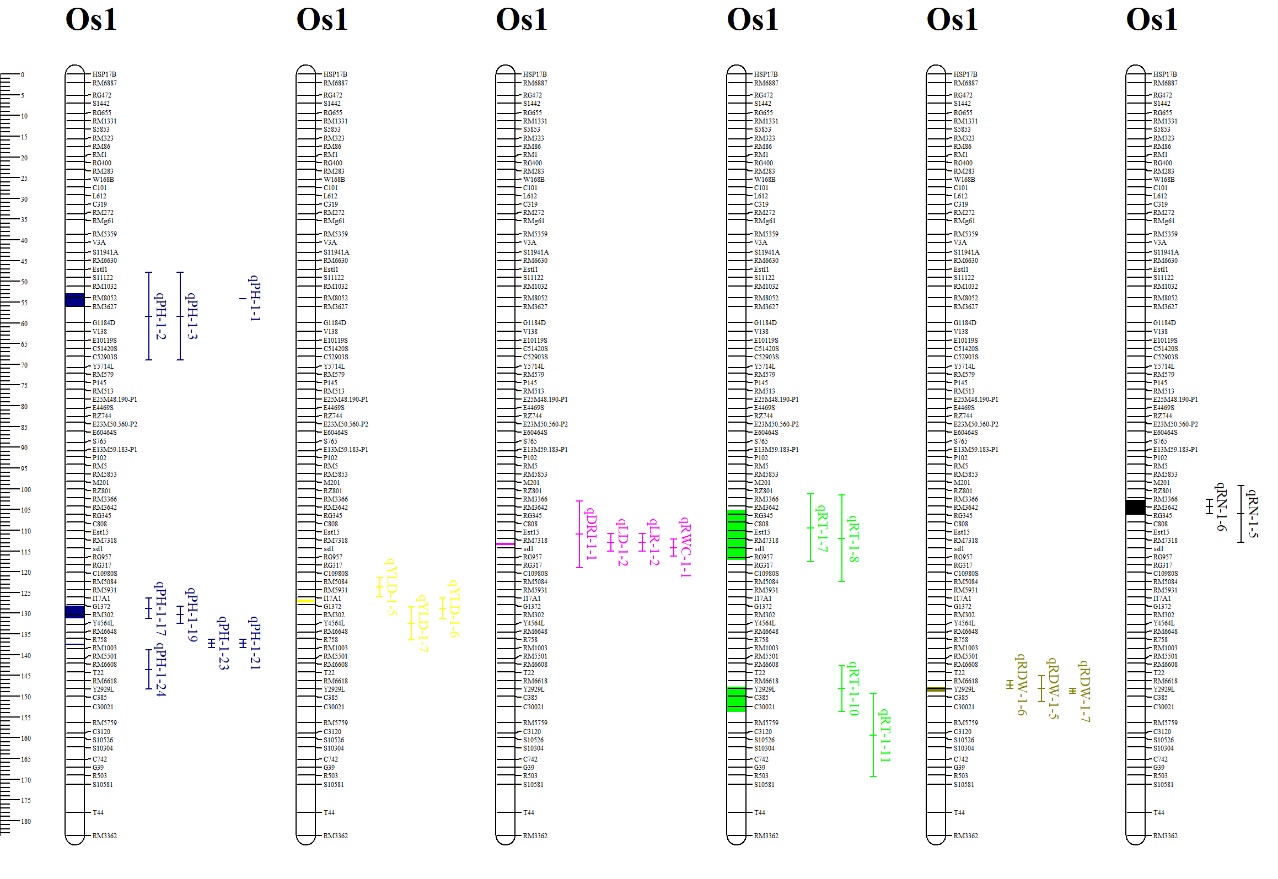

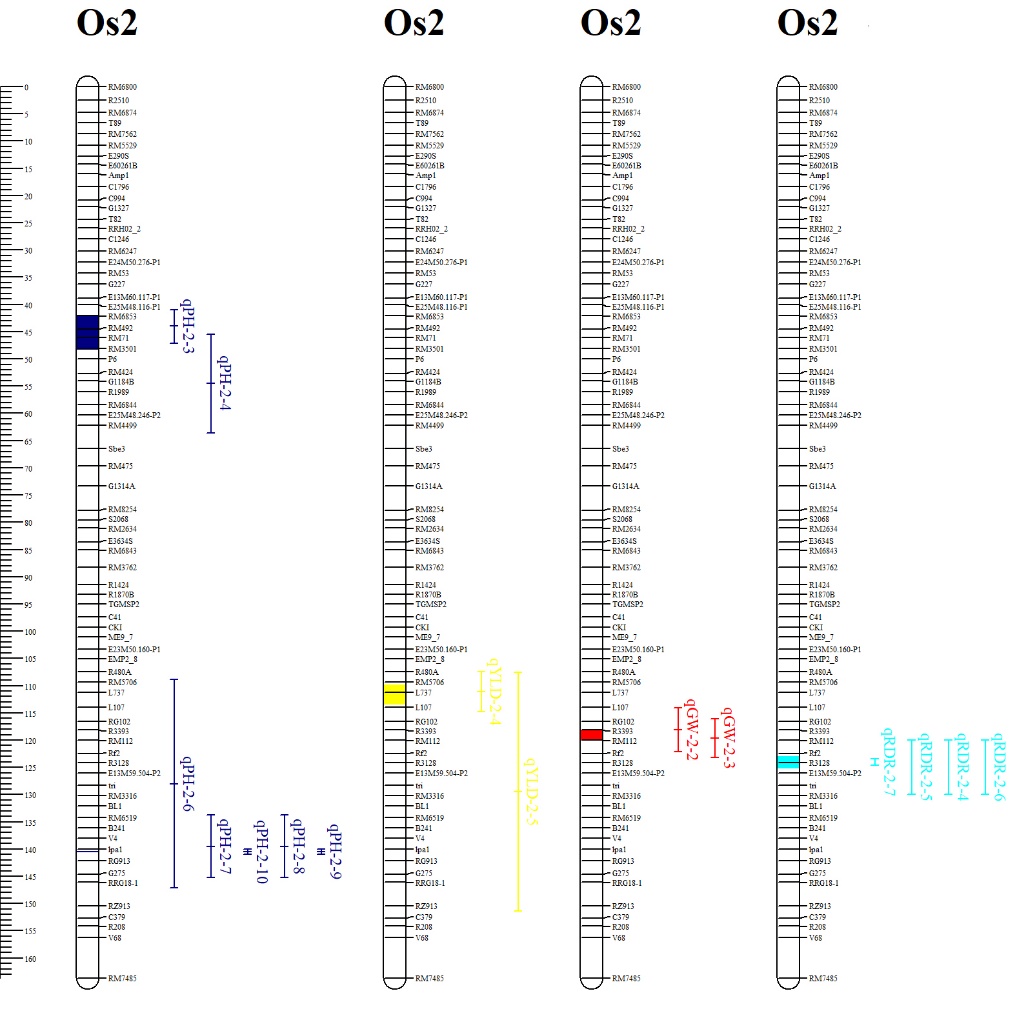

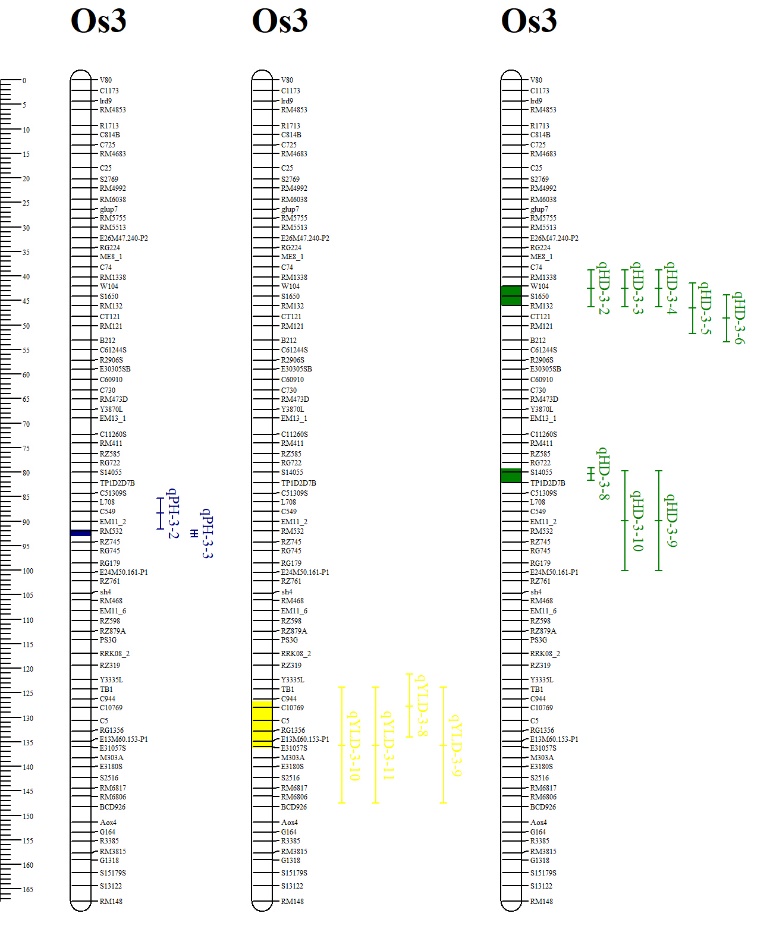

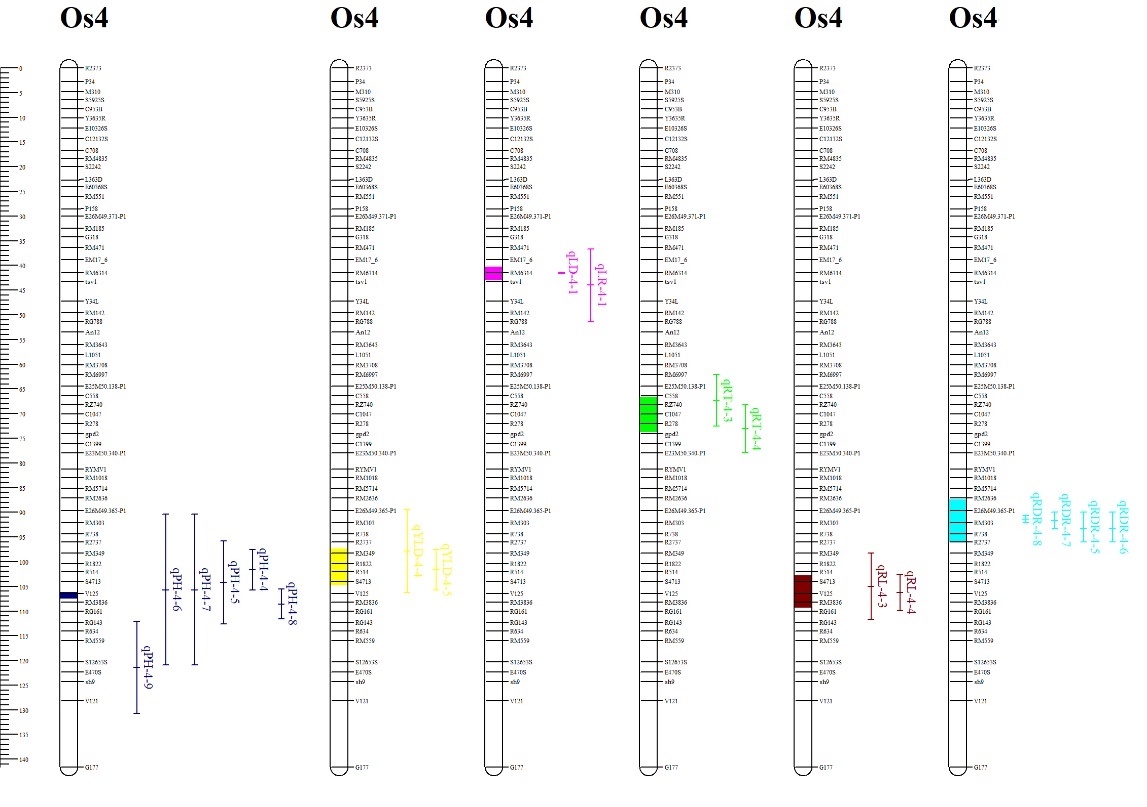

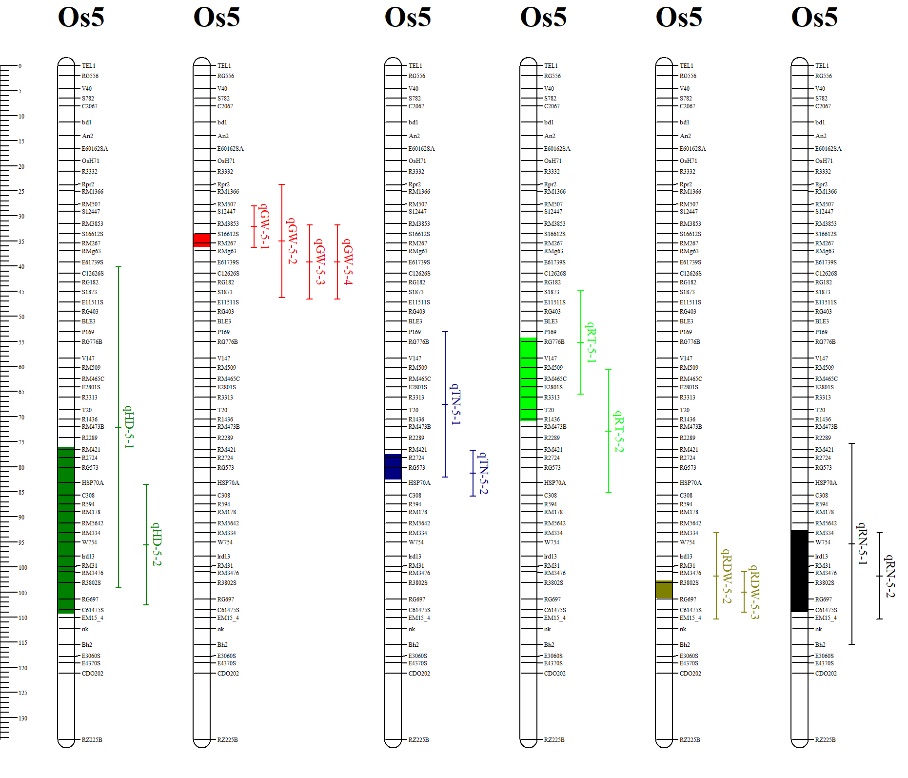

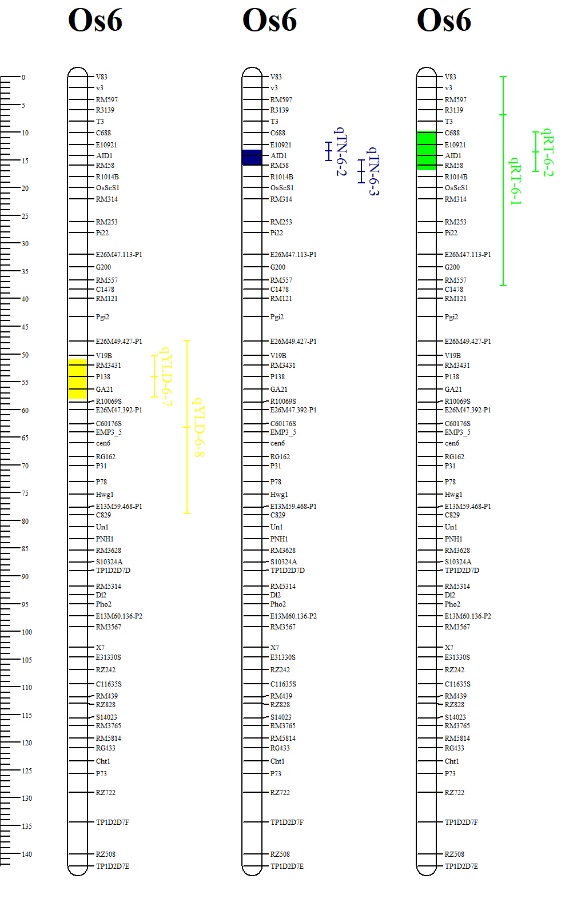

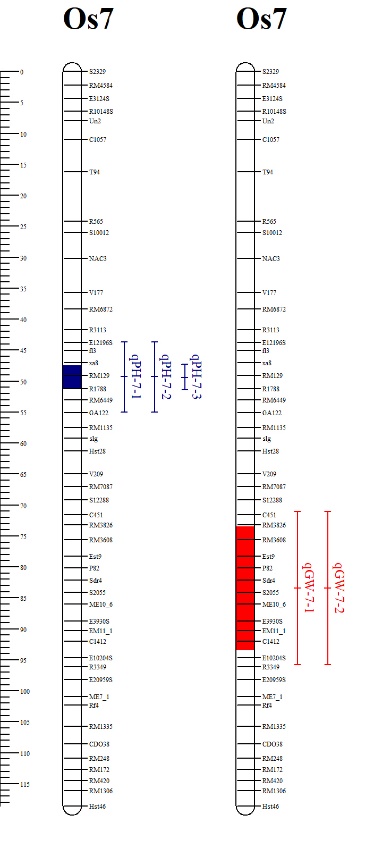

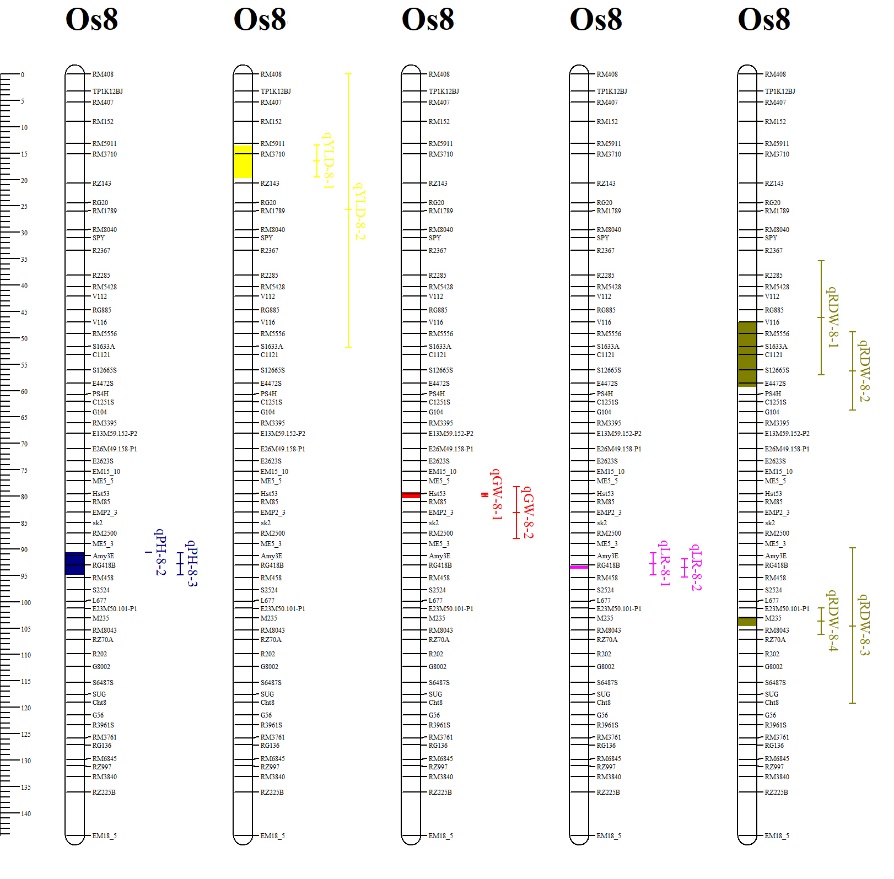

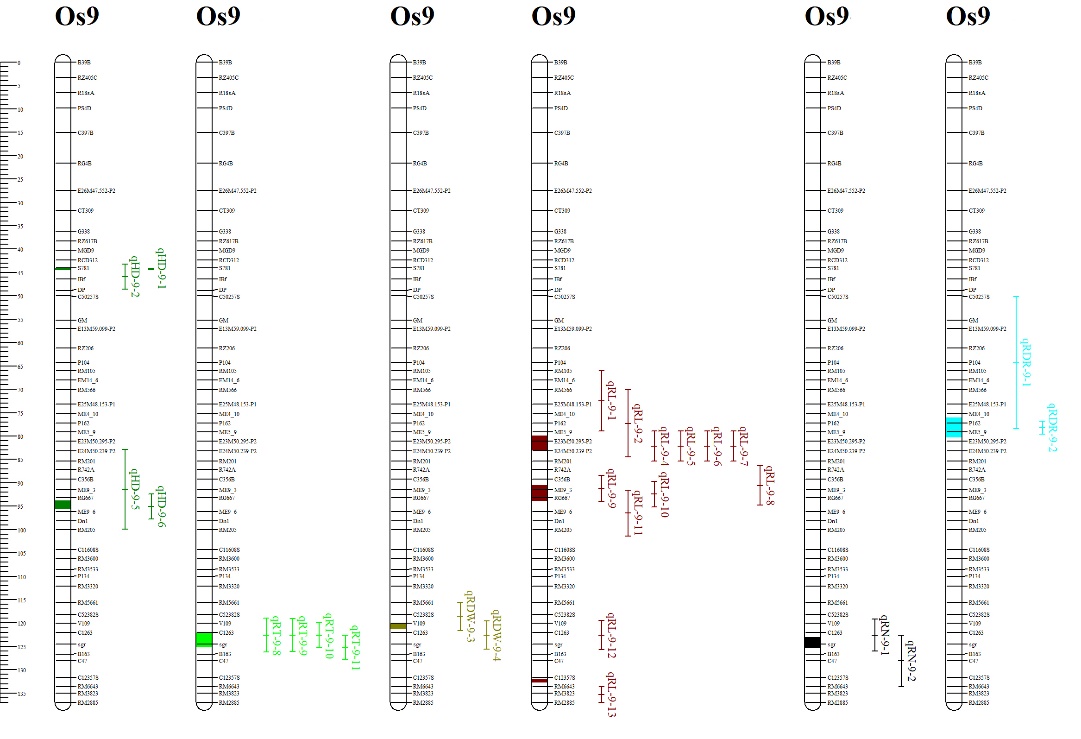

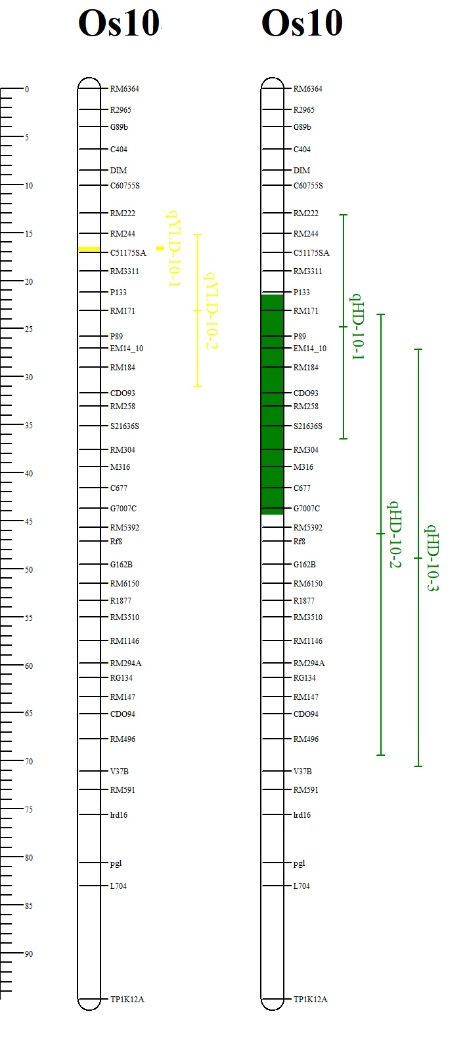

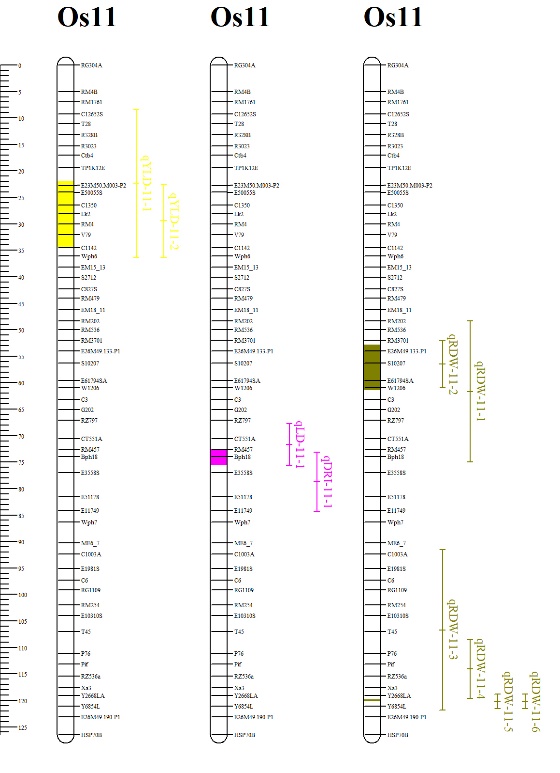

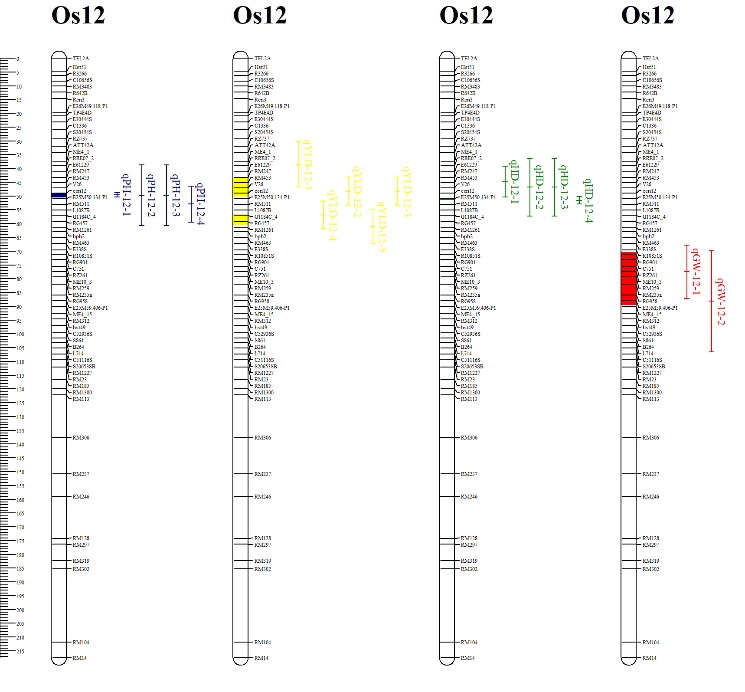


Fig. S1. The chromosomal location of MQTLs and initial QTLs for GW, HD, PH, YLD, TN, DT, RT, RL, RDR, RDW and RN on 12 chromosomes of rice. MQTLs are shown on each chromosome and the lines on the right side of chromosomes indicate the CI of initial QTLs with 95% confidence intervals. Each color represent a specific trait; GW, HD, PH, YLD, DT, RT, RL, RDR, RDW and RN are presented in red, dark green, dark blue, yellow, violet, light green, crimson, light blue, brown and black, respectively. On chromosomes 5 and 6, due to the absence of PH MQTLs, the dark blue indicated the TN. The marker are shown on the right side of chromosomes. The genetic distance (cM) is indicated on the left side of each chromosome.
